# Salvage Ablative Radiotherapy for Isolated Local Recurrence of Pancreatic Adenocarcinoma following Definitive Surgery

**DOI:** 10.3390/jcm13092631

**Published:** 2024-04-30

**Authors:** Edward Christopher Dee, Victor C. Ng, Eileen M. O’Reilly, Alice C. Wei, Stephanie M. Lobaugh, Anna M. Varghese, Melissa Zinovoy, Paul B. Romesser, Abraham J. Wu, Carla Hajj, John J. Cuaron, Danny N. Khalil, Wungki Park, Kenneth H. Yu, Zhigang Zhang, Jeffrey A. Drebin, William R. Jarnagin, Christopher H. Crane, Marsha Reyngold

**Affiliations:** Memorial Sloan Kettering Cancer Center, New York, NY 10065, USA; deee1@mskcc.org (E.C.D.); victor.ng@asterahealthcare.org (V.C.N.); cranec1@mskcc.org (C.H.C.)

**Keywords:** pancreatic cancer, A-RT, pancreatic adenocarcinoma, salvage therapy, radiation therapy, ablative radiation, SABR, SBRT, hypofractionated RT, isolated local recurrence

## Abstract

**Introduction:** The rate of isolated locoregional recurrence after surgery for pancreatic adenocarcinoma (PDAC) approaches 25%. Ablative radiation therapy (A-RT) has improved outcomes for locally advanced disease in the primary setting. We sought to evaluate the outcomes of salvage A-RT for isolated locoregional recurrence and examine the relationship between subsequent patterns of failure, radiation dose, and treatment volume. **Methods**: We conducted a retrospective analysis of all consecutive participants who underwent A-RT for an isolated locoregional recurrence of PDAC after prior surgery at our institution between 2016 and 2021. Treatment consisted of ablative dose (BED10 98–100 Gy) to the gross disease with an additional prophylactic low dose (BED10 < 50 Gy), with the elective volume covering a 1.5 cm isotropic expansion around the gross disease and the circumference of the involved vessels. Local and locoregional failure (LF and LRF, respectively) estimated by the cumulative incidence function with competing risks, distant metastasis-free and overall survival (DMFS and OS, respectively) estimated by the Kaplan–Meier method, and toxicities scored by CTCAE v5.0 are reported. Location of recurrence was mapped to the dose region on the initial radiation plan. **Results**: Among 65 participants (of whom two had two A-RT courses), the median age was 67 (range 37–87) years, 36 (55%) were male, and 53 (82%) had undergone pancreaticoduodenectomy with a median disease-free interval to locoregional recurrence of 16 (range, 6–71) months. Twenty-seven participants (42%) received chemotherapy prior to A-RT. With a median follow-up of 35 months (95%CI, 26–56 months) from diagnosis of recurrence, 24-month OS and DMFS were 57% (95%CI, 46–72%) and 22% (95%CI, 14–37%), respectively, while 24-month cumulative incidence of in-field LF and total LRF were 28% (95%CI, 17–40%) and 36% (95%CI 24–48%), respectively. First failure after A-RT was distant in 35 patients (53.8%), locoregional in 12 patients (18.5%), and synchronous distant and locoregional in 10 patients (15.4%). Most locoregional failures occurred in elective low-dose volumes. Acute and chronic grade 3–4 toxicities were noted in 1 (1.5%) and 5 patients (7.5%), respectively. **Conclusions**: Salvage A-RT achieves favorable OS and local control outcomes in participants with an isolated locoregional recurrence of PDAC after surgical resection. Consideration should be given to extending high-dose fields to include adjacent segments of at-risk vessels beyond direct contact with the gross disease.

## 1. Introduction

Although the management of pancreatic cancer is evolving, progress has been slow, with survival at 5 years estimated at 10% [1,2]. Complete surgical resection, when possible, is considered the best curative treatment [3]. For 15–20% of patients who present with resectable disease, 5-year overall survival after surgery ranges from 12–20% [4]. Isolated local recurrence has been reported in 28–34% [5,6,7,8,9,10,11] and is a significant cause of late disease-related mortality [12,13].

There is currently no established standard treatment for patients with isolated local or locoregional recurrence [14,15,16]. Local therapy options include resection and (chemo)radiotherapy (CRT) [17,18,19,20,21,22,23]. Surgery is rarely an option due to anatomic constraints. Conventionally fractionated standard CRT regimens to a total dose of 39–60 Gy in 1.8–2.0 Gy per fraction are associated with median disease-free and overall survival (DFS and OS, respectively) of 10–17 months and 10–19 months, respectively [20,21,22,24]. Small series using stereotactic body radiotherapy (SBRT) with standard or dose-escalated fractionation schemes including single fraction doses of 18–24 Gy or 5-fraction regimens to 20–50 Gy have demonstrated a median OS of 8.8–17.0 months [17,25,26,27,28,29].

In addition to SBRT, dose escalation to a biologically effective dose (BED) nearing or exceeding 100 Gy, assuming a standard tumor alpha/beta ratio of 10 (BED10), can be accomplished using a hypofractionated approach to a total dose of 67.5–75 Gy in 15–25 fractions [30]. Treatment to approximately BED10 of 100 Gy, also termed ablative radiotherapy (A-RT), using either SBRT or the hypofractionated approach has recently been shown to be associated with better overall survival than standard-dose RT in the setting of primary disease [31,32]. We sought to evaluate the outcomes of patients treated with A-RT for isolated locoregional recurrence and examine the relationship between the subsequent patterns of local progression, radiation dose, and treatment volume.

## 2. Methods

### 2.1. Patient Cohort

This is an institutional review board-approved (16–370) retrospective analysis of all consecutive patients who underwent A-RT for an isolated local or locoregional recurrence after prior pancreaticoduodenectomy or distal pancreatectomy at Memorial Sloan Kettering Cancer Center between June 2016 and 2021. A radiographic/clinical or pathologic diagnosis of recurrence was allowed.

### 2.2. Ablative Technique

The A-RT technique has been described previously [30,33,34]. Briefly, patients were simulated in a customized immobilization device using the Varian RPM system for motion management: deep-inspirational breath hold (preferred) or end-expiratory gating as tolerated by the patient. Target and organ-at-risk (OAR) volumes were delineated on a thin-slice pancreatic protocol contrast-enhanced CT. Prescription doses/fractionation schemes were based on the distance from the target to the luminal GI tract and included 75 Gy/25 fractions (BED10 = 97.5 Gy), 67.5 Gy/15 fractions (BED10 = 97.88 Gy), 60 Gy/10 fractions (BED10 = 96.0 Gy), and 50 Gy/5 fractions (BED10 = 100.0 Gy). Prophylactic low-dose (BED10 < 50 Gy) elective nodal and neural plexus coverage included peripancreatic, celiac axis, and superior mesenteric artery regions in all patients as well as porta hepatis/splenic hilum coverage in select patients depending on the tumor location; in general, 1 cm expansions around vessels were used. Luminal OAR Dmax, D2cc, and D5cc constraints were prioritized over PTV/GTV coverage. Treatments were delivered using daily cone-beam CT (CBCT) guidance. Adaptive re-planning was implemented selectively for patients with consistently unfavorable luminal OAR displacement toward the target on daily CBCTs.

### 2.3. Study Definitions

Local and regional failures were defined according to modified RECIST 1.1 criteria. That is, local in-field failure required an increase of at least 20% in the sum of the largest diameters of target-irradiated lesions or the smaller diameters of pathologic irradiated nodes, along with an absolute increase of at least 5 mm. Regional nodal failure was defined as nodal failure that was not an initially targeted lesion. The following lymph node basins were considered regional (rather than metastatic): common bile duct, common hepatic artery, portal vein, right gastric/pyloric vein, posterior/anterior pancreaticoduodenal vasculature, superior mesenteric vein, and/or right lateral wall of the superior mesenteric artery, common hepatic artery, celiac axis, splenic artery, and/or splenic hilum. Para-aortic nodal basins were not considered regional failures (i.e., defined as metastatic failures).

The pattern of failure was characterized by the location of the recurrence with regard to the dose region on the initial radiation plan. Failures centered within the volume encompassed by the tumor prescription isodose line were defined as high-dose failures while those centered outside the tumor prescription isodose line but within the elective isodose line were defined as low-dose failures. Any lesion within the low-dose region was defined as a regional failure whether it appeared to be a lymph node or tumor extension along the vessel. Out-of-field failure was defined as any local or regional failure with its center outside of the elective low-dose prescription field.

Toxicities were graded based on CTCAE v.5 criteria. Toxicities were defined as acute and late if they occurred within or beyond 3 months of RT initiation, respectively. Attribution of adverse events to RT required confirmed or possible location within the RT field.

### 2.4. Statistical Analysis

In-field local failure (LF), locoregional failure (LRF), distant metastasis-free survival (DMFS), and overall survival (OS) were defined from the diagnosis of recurrence and from the start of RT. For LF, LRF, and DMFS, observations were censored at the date of last evaluation for the event of interest. For OS, patients were censored at the date of last contact. Kaplan–Meier methodology was used to estimate OS and DMFS, while cumulative incidence functions with competing risks of death were used to estimate LF and LRF.

The associations between baseline characteristics and outcomes (OS and LRF from RT start date) were analyzed using Cox proportional hazards regression and Fine–Gray competing risks regression. 

All analyses were conducted using R version 4.2.2 (Vienna, Austria) with the tidyverse (v1.3.2), gtsummary (v1.6.2), tidycmprsk (v0.2.0), and ggsurvfit (v0.2.0) packages.

## 3. Results

### 3.1. Baseline Characteristics

Between June 2016 and January 2021, 65 participants received 67 A-RT courses for isolated local (n = 48) or locoregional recurrence (n = 17) of pancreatic cancer (Table 1). Median age at the time of RT was 67 (range 37–87) years and 36 participants (55%) were male. The primary surgery was pancreaticoduodenectomy (Whipple) for 53 (82%) participants and distal pancreatectomy for 12 (18%) participants. At the time of surgery, 57 (88%) participants had pT1/T2 and 42 (65%) participants had pN+ disease. Margins were negative (R0) in 34 (52%) participants, close (R0, tumor < 1 mm from surgical margin) in 14 (22%) participants, and positive (R1) in 17 (26%) participants. The median disease-free interval to diagnosis of recurrence was 16 months (range, 6–71 months). Median CA 19-9 at the time of recurrence diagnosis and following chemotherapy was 65 U/mL (range, 0–3919 U/mL) and 50 U/mL (IQR 0–561 U/mL), respectively.

Of 65 participants, 27 (42%) received chemotherapy prior to A-RT, including modified FOLFIRINOX (folinic acid, fluorouracil, irinotecan hydrochloride, and oxaliplatin; n = 14), gemcitabine/Nab-paclitaxel (n = 12), and other regimens (n = 2), for a median of 3.2 months (range, 0.5–14.0 months). Participants who did and did not receive chemotherapy began A-RT at a median of 4.2 months (range, 2–14.7 months) and 1.3 months (range, 0.2–6.1 months) after recurrence diagnosis, respectively. Most common fractionation schemes were 75 Gy/25 fx (N = 37) and 67.5 Gy/15 fx (N = 23). Full treatment details are summarized in Table 1.

### 3.2. Disease Outcomes

**Figure 1:**
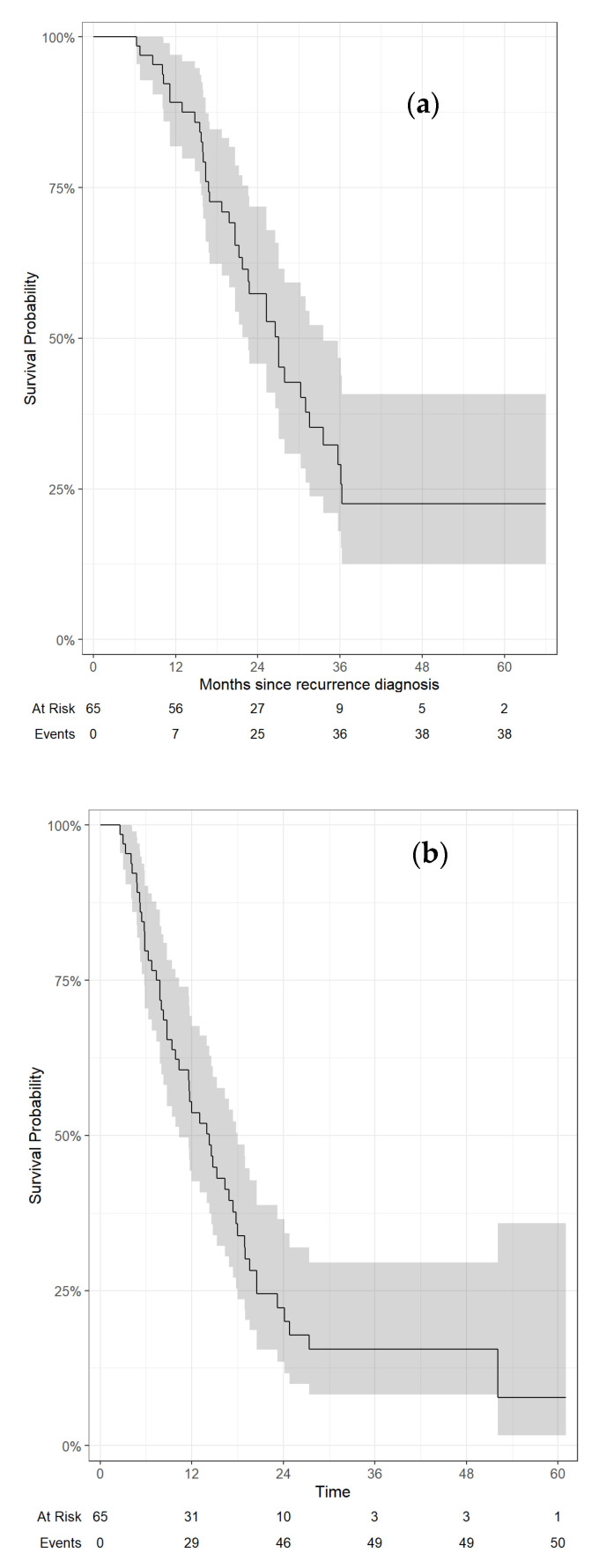
Overall survival (**a**) and distant metastasis-free survival (**b**) from diagnosis of recurrence.

With a median follow-up of 21.0 months from diagnosis of recurrence (and 18.4 months from A-RT start), 38 of 65 (58.5%) patients had died. Median OS rates from diagnosis of recurrence and A-RT were 27 months (95%CI, 23–34 months) and 22 months (95%CI, 18–30 months), respectively. Twelve- and 24-month OS rates from diagnosis of recurrence were 89% (95%CI, 82–97%) and 57% (95%CI, 46–72%), respectively (Figure 1a). Twelve- and 24-month OS rates from A-RT were 81% (95%CI, 72–91%) and 45% (95%CI, 33–61%), respectively.

Median DMFS from diagnosis of recurrence was 14 months (95%CI, 10–18 months); median DMFS from A-RT was 10 months (95%CI, 7.5–14 months). Twelve- and 24-month DMFS rates from diagnosis of recurrence were 54% (95%CI, 43–68%) and 22% (95%CI, 14–37%) (Figure 1b), respectively. Twelve- and 24-month DMFS rates from A-RT were 42% (95%CI, 31–56%) and 18% (95%CI, 10–33%), respectively. 

**Figure 2:**
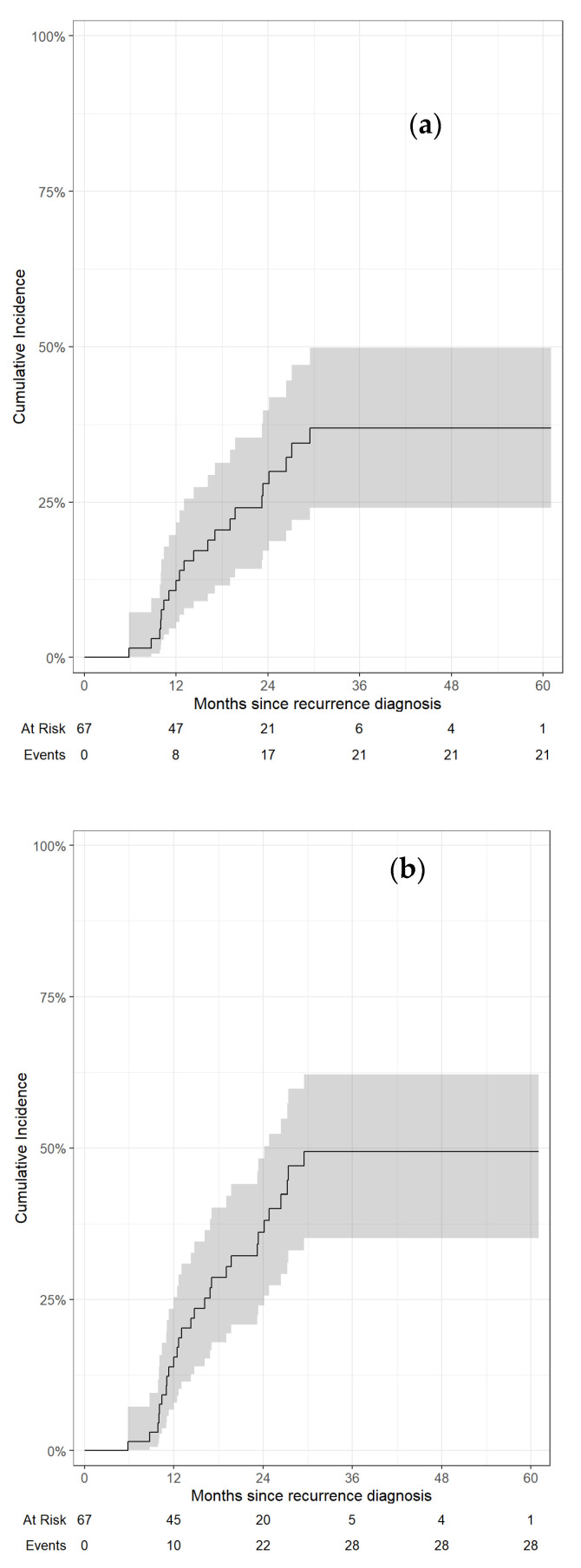
Local in-field failure (**a**) and cumulative locoregional failure (**b**) from diagnosis of recurrence.

Twenty-eight total locoregional failures were recorded, including 13 in-field failures of the irradiated target alone. Twelve- and 24-month rates of in-field LF from diagnosis of recurrence were 12% (95%CI, 5.7–22%) and 28% (95%CI, 17–40%), respectively, while 12- and 24-month rates of total LRF from diagnosis of recurrence were 15% (95%CI, 7.9–25%) and 36% (95%CI, 24–48%), respectively. (Figure 2). Twelve- and 24-month rates of in-field LF from A-RT were 16% (95%CI, 8.0–26%) and 32% (95%CI, 21–35%), respectively. Twelve- and 24-month rates of total LRF from A-RT were 20% (95%CI, 11–31%) and 44% (95%CI 31–56%), respectively. 

### 3.3. Patterns of Failure

**Figure 3:**
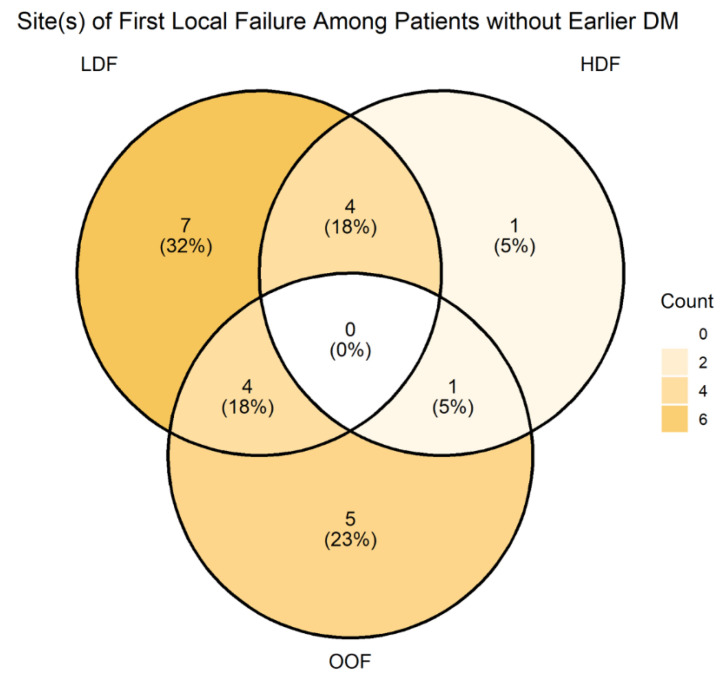
First locoregional failures by RT dose (LDF, low-dose field; HDF, high-dose field; OOF, out-of-field).

The site of first progression after A-RT was distant in 35 patients (53.8%), locoregional in 12 patients (18.5%), and synchronous distant and locoregional in 10 patients (15.4%). Of the 22 first locoregional failures, 17 occurred at least partly in field (i.e., covered by either ablative or elective dose volumes) and 5 were entirely out of field (Figure 3). Of the five isolated out-of-field failures, all were located within 1 cm of the RT field edge, two were in the left para-aortic basin, one was in the gastrohepatic ligament basin, and two were along SMA/SMV just inferior to the treated recurrence.

### 3.4. Predictors of OS and LRF

In the univariable analysis of baseline factors, only reduction in CA 19-9 after chemotherapy was significantly associated with LRF (HR = 0.93; 95%CI, 0.89–0.97, *p* = 0.002) (Table 2). No clinical factors tested, including stage of the primary tumor, time to recurrence, margin status, or receipt of chemotherapy for recurrence were significantly associated with OS after A-RT (Table 2).

### 3.5. Adverse Events 

Of 67 treatment courses among 65 participants, only one case of acute grade 3 toxicity (fatigue) was noted (Table 3). The most common acute toxicity (all grade ≤ 2) was nausea (58.2%), followed by fatigue (55.2%), and diarrhea (34.3%). 

In the sub-acute setting, four (6.0%) cases of grade 3 and one (1.5%) case of grade 4 gastrointestinal toxicity were noted. Grade 4 gastrointestinal bleeding occurred in a participant who received two A-RT treatments one year apart. In addition, one patient died of gastrointestinal bleeding that occurred outside of the irradiated field and was adjudicated to be unrelated to radiation.

## 4. Discussion

Isolated local and locoregional recurrences are common in patients with resected PDAC and appear to benefit from local therapy [19,23]. Herein, we demonstrate that similar to the primary setting, a novel A-RT approach delivering BED10 of 98–100 Gy achieved favorable local control and OS with a low risk of treatment-related morbidity in the salvage setting.

In this very selected cohort, the median OS of 27 months and 2-year 57% OS rate from diagnosis of recurrence compared favorably to those reported in cohorts that underwent re-resection [17]. For instance, Strobel et al. reported on 41 patients who underwent re-resection, with a median survival from reoperation of 26 months [23]. Miyazaki et al. reported on 11 patients who had a median OS after re-resection of 25 (3–61) months [19]. It is important to note that only a small minority of patients with local recurrence—estimated at 2%—are candidates for surgical salvage [17]. While prior surgery may complicate eligibility for salvage surgery, it may actually facilitate delivery of ablative radiation doses in some cases by removing or moving the luminal GI tract farther away from the origin of SMA and celiac axis where these recurrences are often seen. This may contribute to a slightly lower risk of bleeding in this cohort compared to patients treated in the primary setting [33]. The feasibility of A-RT coupled with low rates of grade 3 or higher toxicity make it a viable option in a larger proportion of patients with isolated local recurrence compared to surgery.

With regard to other radiotherapy options, A-RT demonstrates superior outcomes compared to standard-dose (BED10 53–72 Gy) conventionally fractionated CRT or standard-dose SBRT [21,24,25,27]. An older series of patients who underwent chemoradiotherapy demonstrated a median OS from time of treatment ranging from 10 months to 19 months [21,24]. The largest retrospective analysis of 51 patients using 5-fraction SBRT to a total dose of 25–33 Gy showed a median OS and local progression-free survival of 16 and 10 months, respectively [35]. Perhaps the most robust evidence comes from a prospective phase 2 trial in a locally recurrent population (with KRAS mutation and PD-L1 positivity), which randomized 170 participants to receive 5-fraction SBRT to 35–40 Gy (BED10 59.5–72 Gy) with either immunotherapy or gemcitabine, and showed a median OS of 12.8–14.9 months [28]. Subsequent dose–response analysis that dichotomized patients to BED10 of 60–65 Gy vs. > 65 Gy showed a possible association with PFS in the immunotherapy group but not the chemotherapy group, and no association with OS [36]. The dose–response relationship was also examined in a small cohort of 19 patients from Johns Hopkins treated with standard-dose 5-fraction SBRT to a total dose of 25–33 Gy (median BED10 of 54.8 Gy and range of 37.5–54.8 Gy) with a median OS of 17.1 months [29]. Interestingly, in this analysis, BED10 < 54.8 Gy was associated with lower local progression-free survival (1 year, 25.0% vs. 80.2%, *p* < 0.009) [29]. Collectively, these findings suggest that dose escalation may be beneficial, but they failed to test ablative dosing.

Ablative-dose SBRT was given to some of the patients in a 24-participant retrospective cohort reported by Zeng et al. with a median BED10 of 85.50 Gy (range, 71.4–100 Gy) [27]. However, the wide range of dosing and heterogeneity of the patient population where 21% of participants had distant metastasis complicated interpretation of the 1-year local control of 83% and mOS of 12.2 months.

Ablative-dose RT to BED10 of 98–100 Gy using either the hypofractionated technique or 5-fraction SBRT has shown improved local control and OS outcomes in the primary treatment of locally advanced pancreatic cancer [31,33,37,38,39]. The current series constitutes the first experience using dose-escalated RT for salvage therapy and builds on our prior work showing that, despite a high metastatic potential, localized pancreatic cancer patients benefit from more aggressive local therapy.

There are no consensus volumes for irradiation of local recurrence. Our institutional practice has been to extend the elective prophylactic coverage isometrically around the gross disease as well as along the involved mesenteric vessels, making sure to include the origin of the involved mesenteric arteries thought to contain the neural plexi mediating pancreatic cancer spread. Interestingly, most locoregional failures occurred in the field encompassed by low-dose elective coverage volume or immediately beyond it, with some, like the para-aortic basin, within regions that may have been included by the Radiation Therapy Oncology Group 0848 contouring atlas created for the adjuvant setting [40]. This suggests that locoregional failures are predictable and could be more effectively addressed with larger volumes receiving a higher dose of RT. We have now changed our institutional practice to asymmetrically extend the high-dose clinical target volume beyond the gross disease to cover at least 0.5–1.0 cm of the length of any involved mesenteric vessels and the origin of arteries whenever normal tissue constraints allow. Furthermore, a higher microscopic dose exceeding BED10 of 50 Gy may be needed to improve local control. Finally, coverage of para-aortic basins or selective coverage of adjacent non-regional basins (i.e., lymph nodes around the lesser curvature of the stomach for a celiac recurrence) with elective volume may also be considered.

## 5. Limitations

Our findings are limited by the retrospective nature, highly selected single-institution cohort, and relatively small sample size of our study. However, the potential benefit compared to historical results is compelling and consistent with findings in the primary setting. Thus, these findings support further exploration of the role of A-RT for locally recurrent pancreatic adenocarcinoma. 

## 6. Future Directions

Although A-RT achieves favorable local control for patients with ILR, rates of in-field local failure nonetheless underscore the need for further improvement. From a dosimetric and treatment planning standpoint, our institutional practice has evolved to include the origin of the involved mesenteric vessels to target neural plexi that may harbor microscopic disease. However, ablative doses are constrained by the dose tolerances of nearby OARs. Newer technologies, such as MR-guided RT systems, facilitate daily adaptive planning to manage movement of luminal gastrointestinal organs [38]. 

Another consideration is our evolving understanding of the biology of PDAC. The tumor microenvironment (TME) consists of cellular elements (such as cancer-associated fibroblasts and tumor-associated neutrophils) and molecular structures (such as the extracellular matrix) that surround PDAC cells [41]. The TME may promote radiation resistance through factors including an immune-inhibitory environment, increased numbers of mitochondria associated with cancer-associated fibroblasts, and increased tumor hypoxia [41]. Therefore, efforts to target the TME have been explored in PDAC, although success in modulating stromal desmoplasia and immunosuppressive pathways has been limited [42]. Efforts to synergize interventions targeting the stroma and immune elements are under exploration [42]. Furthermore, how radiation can be leveraged to disrupt the TME [43] and potentiate systemic treatments—beyond the integration of RT with immunotherapy—should be explored further.

## 7. Conclusions

In this single-institution series of 65 participants with isolated locoregional recurrence of PDAC after prior resection, we report that A-RT to BED10 of 98–100 Gy achieves favorable local control and overall survival outcomes compared to historical controls treated with surgical resection or standard-dose conventionally fractionated RT or SBRT. Consideration should be given to expanding high-dose clinical target volumes to include adjacent segments of at-risk vessels beyond contact with the gross disease whenever normal tissue constraints allow. In addition, increasing the dose to elective clinical target volume where feasible and selective inclusion of adjacent non-regional nodal basins in the low-dose clinical target volume should be routinely considered. Prospective studies examining A-RT for locally recurrent PDAC are warranted.

## Figures and Tables

**Table 1:** (**a**) Baseline characteristics of the study cohort, (**b**) Treatment details for patients in the study cohort.

(a) Baseline Characteristics of the Study Cohort	
Characteristic	N = 65 *
**Age in years (range)**	67 (37–87)
**Sex**	
Female	29 (45%)
Male	36 (55%)
**Histology**	
Adenocarcinoma	62 (95%)
Mucinous	1 (2%)
Acinar cell	1 (2%)
Colloid	1 (2%)
**T stage**	
T1	16 (25%)
T2	41 (63%)
T3/T4	8 (12%)
**N stage**	
N0	23 (35%)
N1	26 (40%)
N2	16 (25%)
**Initial pathologic stage**	
I	20 (31%)
II	28 (43%)
III	17 (26%)
**Surgery type**	
Distal	12 (18%)
Whipple	53 (82%)
**Margin status at surgery**	
Negative (R0)	34 (52%)
Positive (R1)	17 (26%)
Close (R0, tumor < 1 mm)	14 (22%)
**Disease-free interval, months (range)**	16 (6–71)
**(b) Treatment Details for Patients in the Study Cohort**	
**Characteristic**	N = 65 *
**NACT**	27 (42%)
**NACT regimen**	
FOLFIRINOX	13 (48%)
gem/Nab-paclitaxel	12 (43%)
FOLFOX	1 (4%)
G-FLIP	1 (4%)
No NACT	38
**Length of NACT, months (range) (N = 27)**	3.22 (0.46–13.96)
**CA 19-9 at diagnosis, U/mL (range)**	65 (0–3919)
**CA 19-9 post NACT, U/mL (range) (N = 27)**	50 (0–561)
**Change in CA 19-9, (N = 27)**	−3 (−3358, 135)
**Salvage for local vs. nodal recurrence**	
Local	48 (74%)
Local and nodal	10 (15%)
Nodal	7 (11%)
**Prior RT**	10 (15%)
**Ablative RT dose (N = 67 A-RT courses)**	
75 Gy/25 fractions	37 (55%)
67.5 Gy/15 fractions	23 (34%)
Other fractionation schemes	7 (10%)

* Unless otherwise stated. Abbreviations: NACT: neoadjuvant chemotherapy; FOLFIRINOX: folinic acid, fluorouracil, irinotecan hydrochloride, and oxaliplatin; FOLFOX: folinic acid, fluorouracil, and oxaliplatin; G-FLIP: gemcitabine, 5-fluorouracil, leucovorin, and cisplatin; RT: radiation therapy.

**Table 2:** Univariable regression for OS and LRF.

Characteristic	OS ^1^						LRF ^2^				
	N	Event N	HR ^3^	95% CI ^3^	*p*-Value ^4^		N	Event N	HR ^3^	95% CI ^3^	*p*-Value ^4^
**Central tumor high dose**	65	38			0.92		67	28			0.4
No			—	—					—	—	
Yes			1.05	0.41, 2.69					1.55	0.59, 4.10	
**Change in CA 19-9 (divided by 100)**	26	16	1.00	0.93, 1.06	0.91		26	11	0.93	0.89, 0.97	0.002
**Salvage for primary vs. nodal recurrence**	65	38			0.31		67	28			0.056
Primary			—	—					—	—	
Both			1.94	0.82, 4.57					2.04	0.82, 5.03	
Nodal			0.81	0.25, 2.67					2.89	1.10, 7.61	
**NACT**	65	38			0.78		67	28			0.6
No			—	—					—	—	
Yes			0.91	0.48, 1.74					1.19	0.56, 2.51	
**Length of NACT**	27	17	1.02	0.86, 1.21	0.82		27	12	1.06	0.93, 1.22	0.4
**Prior RT**	65	38			0.69		67	28			0.7
No			—	—					—	—	
Yes			1.20	0.50, 2.90					0.78	0.24, 2.53	
**Surgery type**	65	38			0.33		67	28			0.14
Whipple			—	—					—	—	
Distal			0.66	0.28, 1.58					1.86	0.81, 4.28	
**Disease free interval**	65	38	0.98	0.95, 1.01	0.18		67	28	1.00	0.98, 1.02	0.91
**T stage**	65	38			0.63		67	28			0.65
T1			—	—					—	—	
T2			1.21	0.56, 2.60					1.69	0.60, 4.74	
T3/T4			0.78	0.26, 2.34					1.56	0.47, 5.16	
**N stage**	65	38			0.085		67	28			0.2
N0			—	—					—	—	
N1			1.53	0.70, 3.34					0.45	0.19, 1.06	
N2			2.57	1.13, 5.87					0.62	0.24, 1.63	
**Initial pathologic stage**	65	38			0.24		67	28			0.5
I			—	—					—	—	
II			1.46	0.65, 3.28					0.63	0.29, 1.41	
III			2.11	0.89, 5.04					0.61	0.22, 1.69	
**Margin status at surgery**	65	38			0.97		67	28			>0.9
Negative			—	—					—	—	
Positive			1.05	0.48, 2.33					0.92	0.34, 2.49	
Close			1.11	0.50, 2.45					1.10	0.48, 2.50	

^1^ Cox proportional hazards regression, ^2^ Fine–Gray competing risks regression, ^3^ HR = Hazard Ratio, CI = Confidence Interval, ^4^ Global *p*-value.

**Table 3:** Adverse events by CTCAE v.5.

Adverse Event	Grade				
Acute Toxicity	1	2	3	4	5
Nausea	38 (56.7)	1 (1.5)	0	0	0
Fatigue	29 (43.4)	7 (10.4)	1 (1.5)	0	0
Diarrhea	19 (28.4)	4 (6.0)	0	0	0
Anorexia	17 (25.4)	1 (1.5)	0	0	0
**Subacute toxicity**	**1**	**2**	**3**	**4**	**5**
Gastrointestinal toxicity			4 (6.0)	1 (1.5)	1 (1.5)

Percentages reported out of 67 treatment courses among 65 patients.

## Data Availability

The data that support the findings of this study are available from the corresponding author upon reasonable request.
